# Ag Loading Enhanced Photocatalytic Activity of g-C_3_N_4_ Porous Nanosheets for Decomposition of Organic Pollutants

**DOI:** 10.3389/fchem.2019.00091

**Published:** 2019-04-02

**Authors:** Kezhen Qi, Yi Li, Yubo Xie, Shu-yuan Liu, Kun Zheng, Zhe Chen, Ruidan Wang

**Affiliations:** ^1^Institute of Catalysis for Energy and Environment, College of Chemistry and Chemical Engineering, Shenyang Normal University, Shenyang, China; ^2^Key Laboratory of Advanced Energy Materials Chemistry (Ministry of Education), Nankai University, Tianjin, China; ^3^Department of pharmacology, Shenyang Medical College, Shenyang, China; ^4^Department of Hydrogen Energy, Faculty of Energy and Fuels, AGH University of Science and Technology, Kraków, Poland; ^5^School of Material Science and Technology, Jilin Institute of Chemical Technology, Jilin City, China

**Keywords:** Ag nanoparticles, Ag/g-C_3_N_4_, photocatalytic activity, organic pollutant, electron-hole separation, modification

## Abstract

The g-C_3_N_4_ porous nanosheets with different loading amount of Ag nanoparticles (NPs) are successfully prepared by a simple liquid-phase reduction method. These Ag/g-C_3_N_4_ composites have an improved photocatalytic performance for decomposing organic pollutants compared with that of pure g-C_3_N_4_ nanosheets. Many measurements have been used for characterizing the samples, such as XRD, FTIR, UV-Vis DRS, PL, XPS, EDS, SEM, and TEM. In Ag/g-C_3_N_4_, the Ag NPs are uniformly coated on the g-C_3_N_4_ surface, the diameter is mainly in the range of 8~18 nanometers. Loading of Ag NPs expand the response to the visible light for g-C_3_N_4_ and increasing the producing rate of photogenerated e^−^-h^+^ pairs. The loading of silver NPs obviously enhances the photocatalytic activity of C_3_N_4_ nanosheets toward the Rhodamine B (RhB) decomposition under the simulated sunlight irradiation. With different loading amounts of Ag NPs, Ag/g-C_3_N_4_ (3 wt% of Ag) showed the highest photocatalytic activity for RhB decomposition among these as-prepared samples, which is 10 times of the rate of pure C_3_N_4_. Based on the experimental results, a possible photocatalytic mechanism for Ag/g-C_3_N_4_ is proposed.

## Introduction

The organic pollutants in waste water has become a serious problem that threatens human health (Zhang and Wen, 2008). Solar energy, as a clean energy, has been widely concerned. It is a very effective way to solve the problem of water pollution by using solar energy (George et al., 2015; Wang et al., 2017, 2018a; Qi et al., 2018a; Shi et al., 2018; Wei et al., 2018; Yan et al., 2018a,b; Zhong et al., 2018). Since Fujishima et al. reported the phenomenon of water splitting for hydrogen production on TiO_2_ photoanode (Fujishima and Honda, 1972), the study of semiconductor photocatalysts has become very popular (Tao et al., 2014; Wei et al., 2015; Wen et al., 2015; Park et al., 2016; Qi et al., 2017b, 2018b; Xia et al., 2017).

The photocatalysis technology has been widely used to treat the organic polluted waste water because of its simpler equipment, convenient operation, energy saving, environmental protection, and strong oxidizing ability. Semiconductor photocatalysts mainly include graphite nitride (g-C_3_N_4_) (Cao et al., 2015), metal oxide (Qi et al., 2017a), and metal sulfide (Hong et al., 2015). Among these materials, g-C_3_N_4_, as an important photocatalyst, has received widely attention, because of its unique characteristics, including metal free, non-toxic, easy preparation, suitable band gap, and cheap (Cao and Yu, 2014). However, the photocatalytic activity of g-C_3_N_4_ is still very low, it is difficult to use in real life, due to the low utilization efficiency of sunlight and the fast recombination of photogenerated e^−^-h^+^ pairs (Martha et al., 2013; Zhu et al., 2018). Up to now, many efforts have been devoted to improve the photocatalytic performance of g-C_3_N_4_ (Xiang et al., 2011; Akple et al., 2015; Xu et al., 2018; Fu et al., 2019). For example, Liu et al. used metal doping to enhance the visible light adsorption of g-C_3_N_4_ and found that it enhanced the photocatalytic activity in the photocatalysis of water splitting for hydrogen production (Niu et al., 2012). Cheng et al. demonstrated that through building heterojunction of ZnO/g-C_3_N_4_, the photocatalytic activity for the decomposition of organic dyes is enhanced (Cheng et al., 2013). Fina et al. reported that loading Pt nanoparticles (NPs) can enhance activity of g-C_3_N_4_ photocatalytic water splitting into H_2_ production (Fina et al., 2015). In these methods, depositing noble metals (Au, Ag, Pt, or Pd) on the g-C_3_N_4_ surface is useful to enhance the photocatalytic activity of g-C_3_N_4_ (Wen et al., 2017; Tong et al., 2018; Wang et al., 2018b). However, the mechanism of interaction between the loading noble metals and C_3_N_4_, and how they work to enhance the photocatalytic activity of g-C_3_N_4_ are limited to know.

This work reports a simple liquid-phase reduction method to prepare the Ag/g-C_3_N_4_ composites. The structure, morphology, optical property, and photocatalytic activity of the as-prepared Ag/g-C_3_N_4_ samples are investigated. The effects of loading content of Ag on the light absorbency and photocatalytic activity of C_3_N_4_ are studied. Under the simulated sunlight, the photocatalytic performance of g-C_3_N_4_ for Rhodamine B (RhB) photodegradation is obviously improved after loading Ag NPs. Finally, a possible photocatalytic mechanism of the Ag/g-C_3_N_4_ composite is given.

## Experimental

### Synthesis

The g-C_3_N_4_ nanosheet was prepared via thermal polycondensation of urea. Fifteen grams urea was placed into a covered ceramic crucible and heated to 500°C for 5 h in air, at the heating rate of 10°C min^−1^. After the reaction, it cooled down to room temperature naturally, the product was collected and grind to powder. The Ag/g-C_3_N_4_ composite was synthesized by a liquid-phase reduction method. First 0.5 g of g-C_3_N_4_ was put in 50 mL of water and ultrasonic treated for 5 min. Second, a certain amount of AgNO_3_ (5 mM) aqueous solution was put into the above solution and maintain stirring. Third, a certain amount of NaBH_4_ [the molar ratio of n(AgNO_3_):n(NaBH_4_) = 1:5] dissolved in 30 mL of water, and then put into the above solution, stirring for 1 h. Following, the product was centrifuged and washed with absolute ethanol and distilled water, respectively. Finally, these samples are dried in vacuum oven at 70°C for 5 h. By varying the amount of using AgNO_3_, a series of samples with different ratios of Ag to g-C_3_N_4_ [m(Ag):n(g-C_3_N_4_) = 1, 2, 3, 4, and 5%] were prepared and labeled as 1%-Ag/g-C_3_N_4_, 2%-Ag/g-C_3_N_4_, 3%-Ag/g-C_3_N_4_, 4%-Ag/g-C_3_N_4_, and 5%-Ag/g-C_3_N_4_, respectively.

### Characterization

The crystal phases of products were studied by X-ray diffraction (XRD) (X-ray diffractometer, Cu Kα, λ = 1.54056 Å) (Bruker D5005, Germany). Fourier transform infrared (FT-IR) spectra were conducted using a Nicolet Magna 560 (US) spectrophotometer. X-ray photoelectron spectroscopy (XPS) was measured on a PHIQ 1,600 XPS (US) instrument. The weight percentages of Ag in the Ag/g-C_3_N_4_ photocatalysts was studied by inductively coupled plasma atomic emission spectrometry (ICP-AES, Shimadzu ICP-7510, Japan). High resolution transmission electron microscopy (HRTEM) was taken by a JEOL JEM-2100F (Japan) electron microscope. UV–vis absorbance spectra were collected on a Shimadzu UV-3100 (Japan) spectrophotometer, using BaSO_4_ as reference. The photoluminescence (PL) spectra of g-C_3_N_4_ and Ag/g-C_3_N_4_ samples were studied on a Varian Cary Eclipse (US) spectrometer equipped with an excitation wavelength of 325 nm.

### Photocatalytic Performance

The photocatalytic activity of pure g-C_3_N_4_ and Ag/g-C_3_N_4_ samples was examined by photodegradation of RhB under the simulated sunlight irradiation, which was obtained from an 500 W Xe lamp. Ten milligram of samples were dispersed in 25 mL of RhB aqueous solution (10 mg/L RhB aqueous solution). Prior to the irradiation, the reaction solution was magnetically stirred in the dark for 30 min to get adsorption-desorption equilibrium for the dyes on photocatalyst surface. During the photocatalytic degradation, 2 mL of the sample was withdrawn from the reaction solution at the time intervals of every 15 min and then centrifuged to remove the particles. Then the concentration of RhB was examined by UV–vis spectrophotometer, at the absorbance wavelength of 553 nm. The photodegradation rate of RhB was calculated by the formula: D = *C/C*_0_ × 100%, where *C*_0_ is the initial concentration of RhB, and *C* is the concentration of RhB at a time *t*.

### Photoelectrochemical Measurement

The photoelectrochemical performance was studied on a CHI 660D electrochemical work station with a standard three-electrode system. Put g-C_3_N_4_ or Ag/g-C_3_N_4_ on the ITO glass surface as the working electrode. A piece of Pt wire and a calomel electrode were used as the counter electrode and reference electrode, respectively. The electrolyte is 0.1 mol/L Na_2_SO_4_ aqueous solution. Five milligram photocatalysts were mixed with 1 mL ethanol and then the mixture was coated on 2 × 4 cm ITO glass for use as an electrode. Electrochemical impedance spectroscopy (EIS) Nyquist plots were conducted at an open current potential with an amplitude of 5 mV and the frequency range was from 10^5^ to 1 Hz.

## Results and Discussion

### XRD Patterns

**Figure 1:**
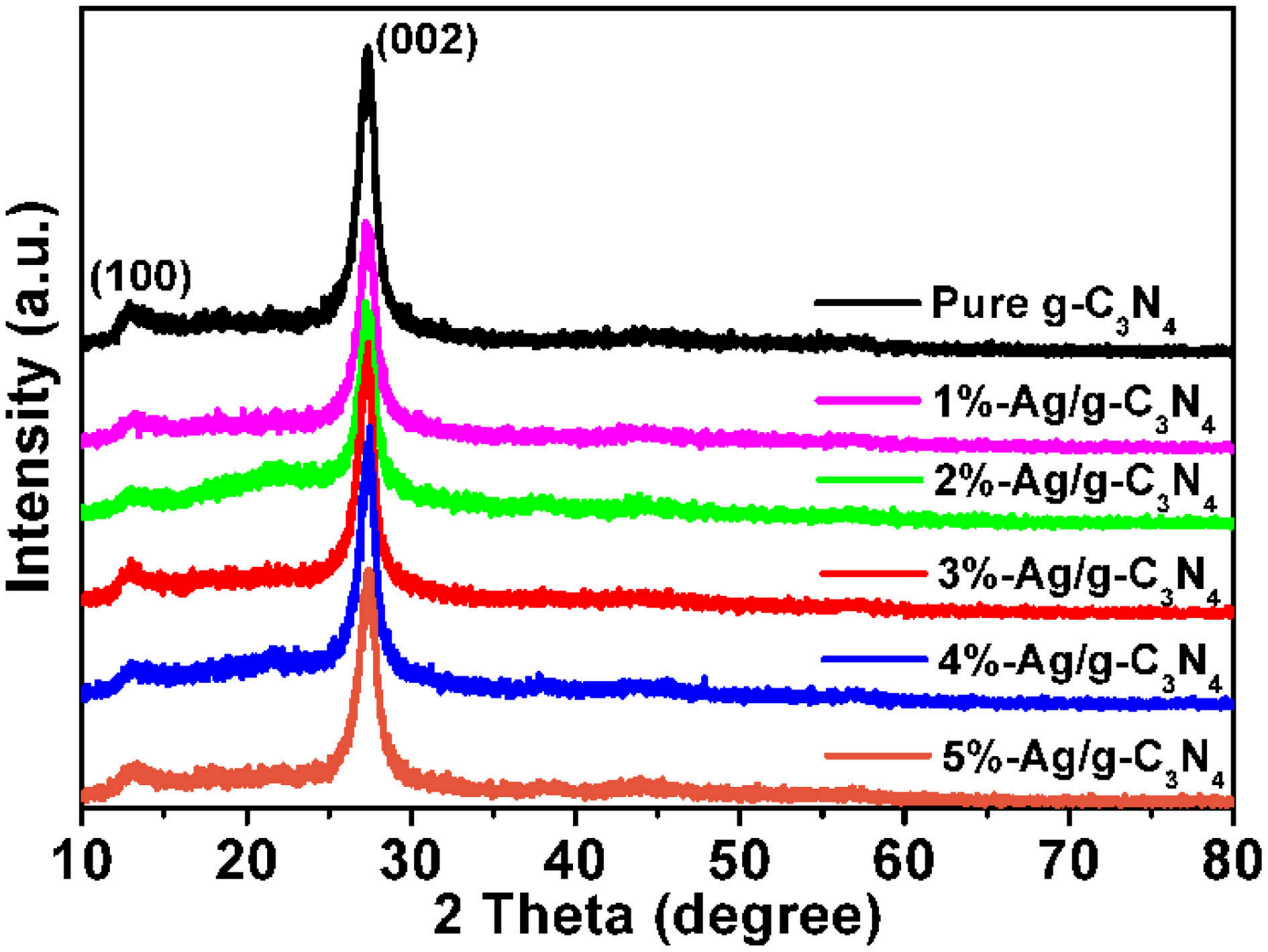
XRD patterns of pure g-C_3_N_4_ and Ag/g-C_3_N_4_ samples.

The crystal phase of as-prepared samples is studied by XRD measurements, and the XRD patterns are shown in Figure 1. The pure g-C_3_N_4_ nanosheets and Ag/g-C_3_N_4_ nanocomposites have two dominant peaks at 13.1° and 27.5°, indexed to g-C_3_N_4_ (JCPDS87-1526) (Yang et al., 2013b). The peak at 27.5° is ascribed to the typical (002) plane with planar distance of 0.33 nm corresponding to interlayer-stacking of aromatic segments. The peak at 13.1° with distance of 0.675 nm is indexed to the (100) plane corresponding to in-plane structural packing (Dong et al., 2011; Liu et al., 2011). Compared with the pure g-C_3_N_4_ nanosheets, the intensity of the diffraction peak at 27.5° becomes weaker with increasing content of loading Ag NPs. The diffraction peak related to Ag NPs is not found, because of the low Ag loading amount and the high dilution effect of Ag NPs on the g-C_3_N_4_ surface (Zhou et al., 2014; Fu et al., 2015). As follows, the XPS and EDS data demonstrate the existence of Ag loading on the g-C_3_N_4_ surface.

### FTIR Analysis

**Figure 2:**
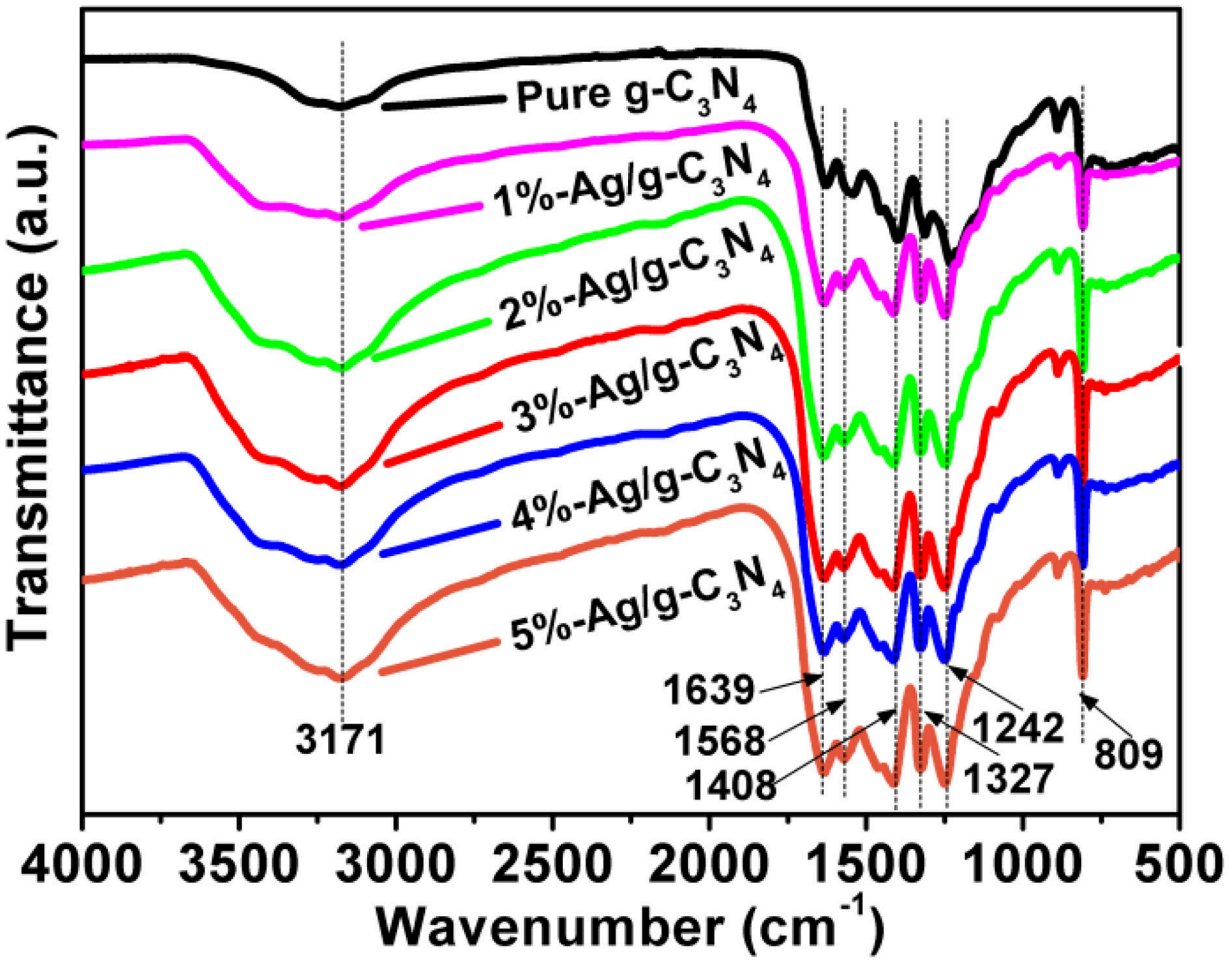
FTIR spectra of pure g-C_3_N_4_ and Ag/g-C_3_N_4_ samples.

The FTIR spectra are similar between the pure g-C_3_N_4_ nanosheets and Ag/g-C_3_N_4_ composites with different Ag loading amounts (Figure 2). The peak at 1,639 cm^−1^ can be ascribed to the stretching vibration of C-N groups, and the peaks at 1,242, 1,327, 1,568 and 1,408 cm^−1^ can be attributed to the aromatic C-N stretching vibration (Aghdam et al., 2017). The peak at 809 cm^−1^ corresponds to the breathing mode of triazine units (Sun et al., 2012). The peak at 3171 cm^−1^ is attributed to the stretching vibration of N-H group (Yang et al., 2013a). All these characteristic FTIR peaks suggest that the overall structure of g-C_3_N_4_ maintains the original form after Ag NPs loading.

### TEM Images

**Figure 3:**
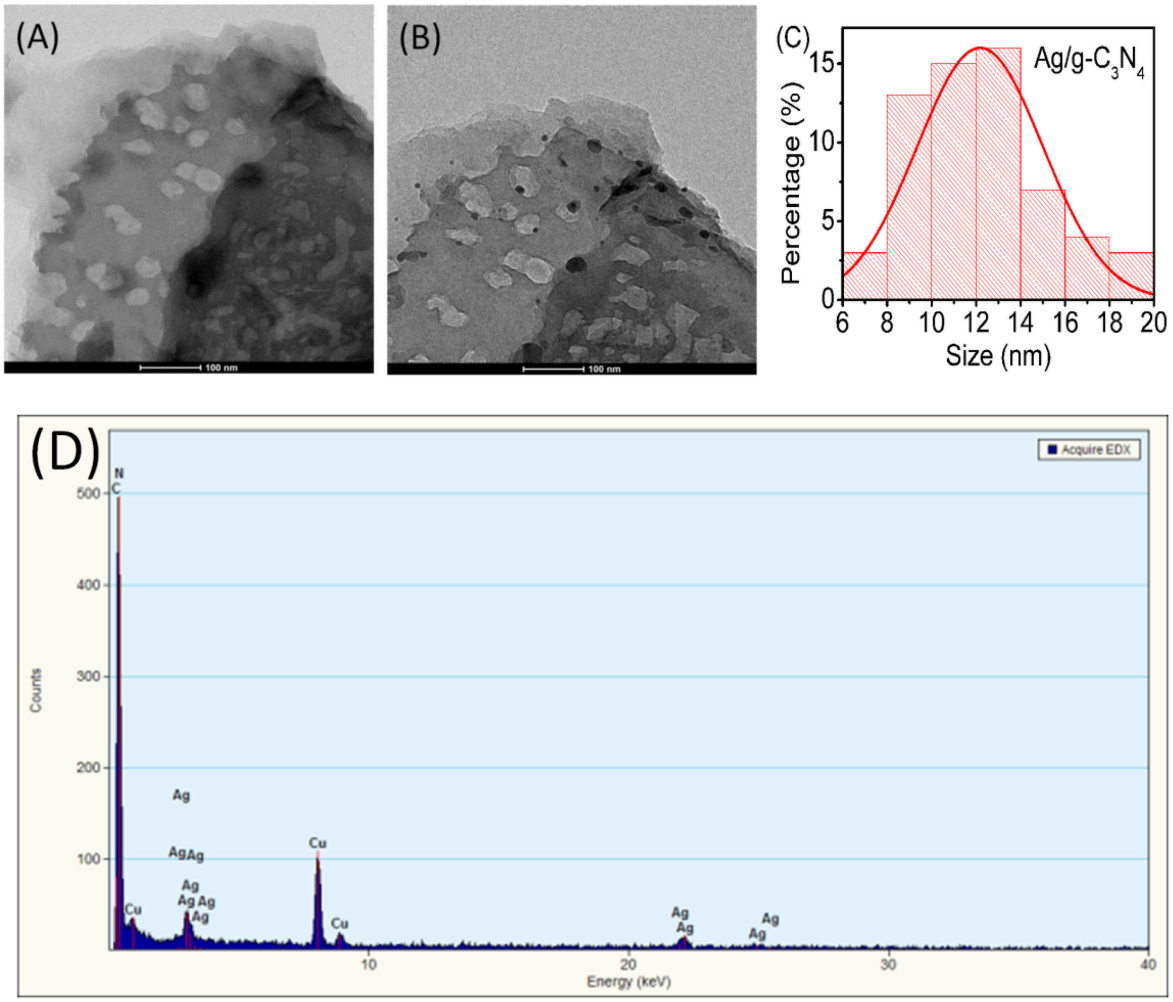
TEM images of **(A)** pure g-C_3_N_4_ and **(B)** 3%-Ag/g-C_3_N_4_. **(C)** size distribution of Ag NPs on 3%-Ag/g-C_3_N_4_. **(D)** EDX mapping of 3%-Ag/g-C_3_N_4_.

The morphology and microstructure of the pure g-C_3_N_4_ and 3%-Ag/g-C_3_N_4_ samples were investigated by TEM measurements. The TEM image of pure g-C_3_N_4_ shows that it is a two-dimensional nanosheet with some holes in the size range of 10–30 nm (Figure 3A). TEM image of the 3%-Ag/g-C_3_N_4_ sample (Figure 3B) shows that Ag NPs, observed as black dots, uniformly disperse on g-C_3_N_4_ surfaces. The size of Ag NPs is from 6 to 20 nm, indicating that these Ag NPs are Ag clusters on the surface of g-C_3_N_4_. The size distribution of Ag NPs on 3%-Ag/g-C_3_N_4_ is presented in Figure 3C, which is mainly in the range of 8–18 nm. The energy dispersive X-ray spectrum (EDS) also confirms that Ag NPs exist on the surface of g-C_3_N_4_ (Figure 3D). Also, it shows that the 3%-Ag/g-C_3_N_4_ sample is consisted of C, N, and Ag elements, which confirms that Ag NPs successfully adsorbed on the g-C_3_N_4_ surface.

### XPS Analysis

**Figure 4:**
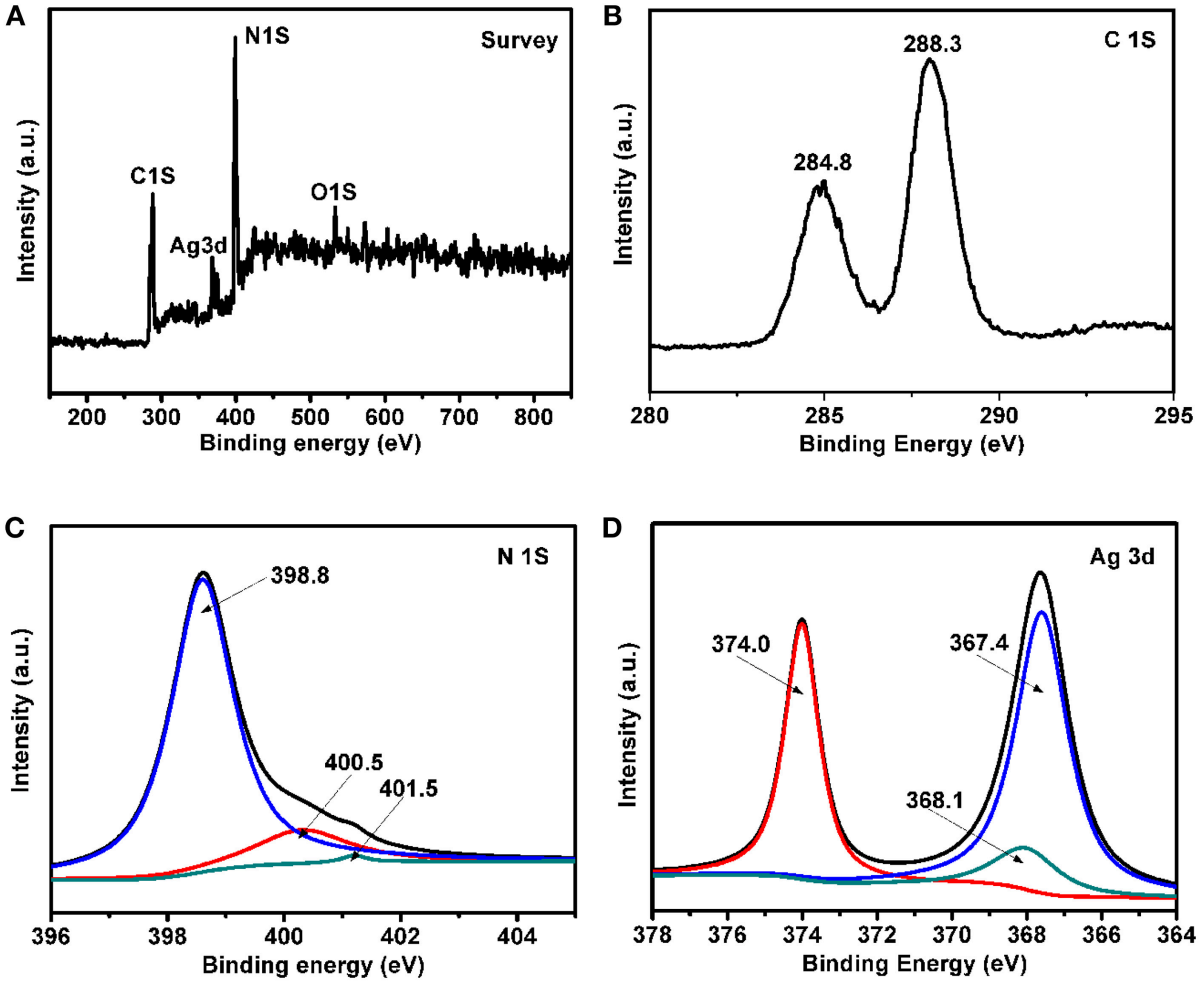
XPS spectra of 3%-Ag/g-C_3_N_4_ composites: **(A)** survey XPS spectrum, high resolution of **(B)** C1s spectra, **(C)** N1s spectrum, **(D)** Ag 3d spectrum.

The surface elemental composition and chemical states of Ag/g-C_3_N_4_ are studied by XPS, here 3%-Ag/g-C_3_N_4_ is selected for study (Figure 4). The elements C, N, O, and Ag are clearly observed in the survey spectrum (Figure 4A). The peak located at 531 eV is assigned to O, which may be the water molecules at the sample surface (Hu et al., 2015). Two C 1 s peaks locate at 284.8 eV and 288.3 eV (Figure 4B). The peak located at 284.8 eV is assigned to sp^2^-hybridized C atoms, and the 288.3 eV peak can be assigned as N-C = N_2_ groups (Wu et al., 2015). As shown in Figure 4C, the peaks of N 1 s locate at 398.8, 400.5, and 401.5 eV, which can be ascribed to sp^2^ bonded nitrogen C-N-C groups, sp^3^ tertiary nitrogen N-(C)_3_ and amino functional groups (C-N-H), respectively (Qi et al., 2019). The spectrum of Ag 3d (Figure 4D) shows that the peaks located at 367.4 and 374.0 eV can be assigned as Ag 3d^5/2^ and Ag 3d^3/2^, respectively (Yang et al., 2016). This confirms that Ag NPs are successfully coasted on g-C_3_N_4_ surfaces. The peak at 368.1 eV is assigned as Ag(I), which indicates the formation of Ag_2_O on the surface of metallic Ag (Tian et al., 2015). The actual content of Ag in the Ag/g-C_3_N_4_ composites was studied by ICP-AES analysis. The result shows that the weight percentages of Ag in 1%-Ag/g-C_3_N_4_, 2%-Ag/g-C_3_N_4_, 3%-Ag/g-C_3_N_4_, 4%-Ag/g-C_3_N_4_, and 5%-Ag/g-C_3_N_4_ were measured to be 0.72, 1.43, 2.09, 2.74, and 3.55, respectively. The measured value by ICP-AES is a little smaller than the theoretical value for the weight percentages of Ag in Ag/g-C_3_N_4_ composites, but both of the two changing trends are the same.

### UV-vis Diffuse Reflectance Spectra

**Figure 5:**
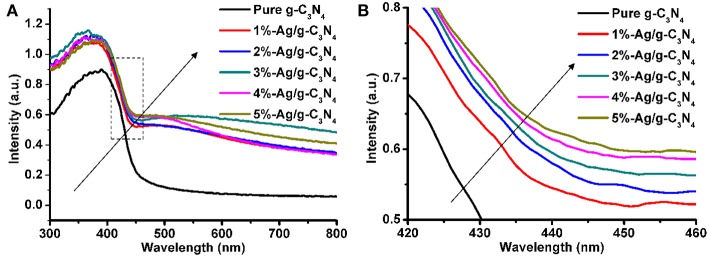
**(A)** UV-Vis diffuse reflectance absorption spectra of the pure g-C_3_N_4_ and Ag/g-C_3_N_4_ samples. **(B)** Magnify the square apart of picture **(A)**.

The UV-DRS measurement is used to study the optical adsorption property of the pure g-C_3_N_4_ nanosheets and Ag/g-C_3_N_4_ composites (Figure 5). Figure 5A shows that the light absorption edge of the pure g-C_3_N_4_ is at 440 nm, which agrees with the intrinsic band gap of bulk g-C_3_N_4_ (Chen et al., 2016). Compared with pure g-C_3_N_4_ nanosheets, Ag/g-C_3_N_4_ composites have an additional weak and broad absorption peak around 450–600 nm, which is characteristic of the silver surface plasmon resonance band (Liu et al., 2013). The Ag/g-C_3_N_4_ composite shows similar light absorption range with that of pure g-C_3_N_4_, but the visible light adsorption is increased, as shown in Figure 5B. Thus, the samples with the increasing of Ag loading amount change the color from yellow to dark gray.

### PL Spectra

**Figure 6:**
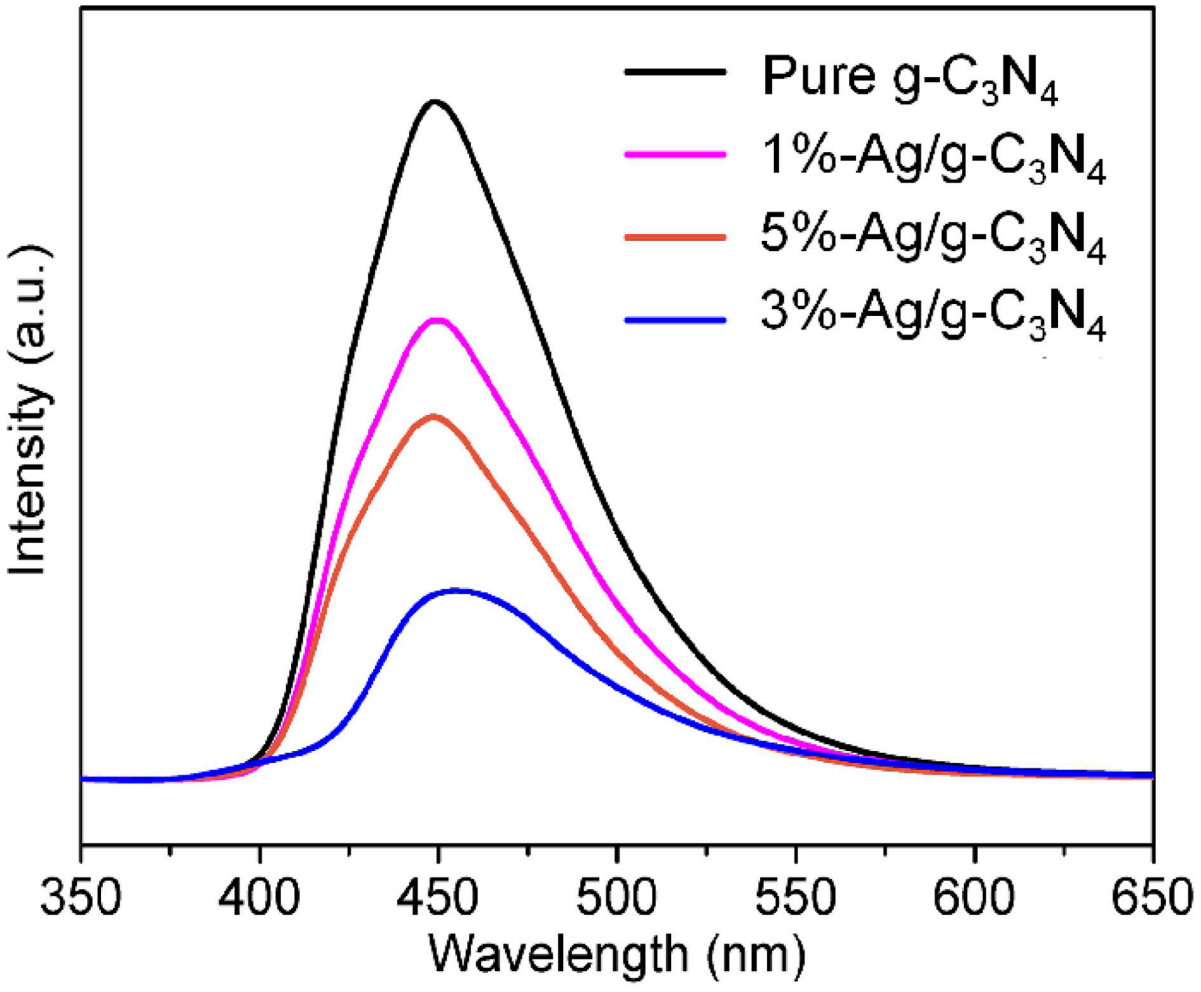
PL spectra of the pure g-C_3_N_4_, 1%-Ag/g-C_3_N_4_, 3%-Ag/g-C_3_N_4_, and 5%-Ag/g-C_3_N_4_ sample.

Photoluminescence measurement is an useful method to analyze the separation efficiency and the life time of photogenerated carriers, as shown in Figure 6. PL spectra of pure g-C_3_N_4_ and Ag/g-C_3_N_4_ are taken by the exciting light of 325 nm. A strong broad peak at ~460 nm is observed. Compared with pure g-C_3_N_4_, the PL intensity of Ag/g-C_3_N_4_ composites decreases significantly. The weaker peak intensity of PL results in a slower recombination rate of photogenerated carriers (Ong et al., 2014). In Ag/g-C_3_N_4_ composites, Ag NPs combine with the g-C_3_N_4_ surface strongly, and effectively reduce the recombination rate of e^−^-h^+^ pairs. This improved separation efficiency of photogenerated carriers leads to increasing e^−^ and h^+^ to join the photocatalytic process of Ag/g-C_3_N_4_. However, over loading of Ag on g-C_3_N_4_, such as 5%-Ag/g-C_3_N_4_, the PL intensity is getting increase again, indicating the increase of the combination of photogenerated carriers. It clearly sees that the Ag/g-C_3_N_4_ composites with proper loading amounts of Ag have a potential for using as photocatalysts with high activity.

### Photocatalytic Activity

**Figure 7:**
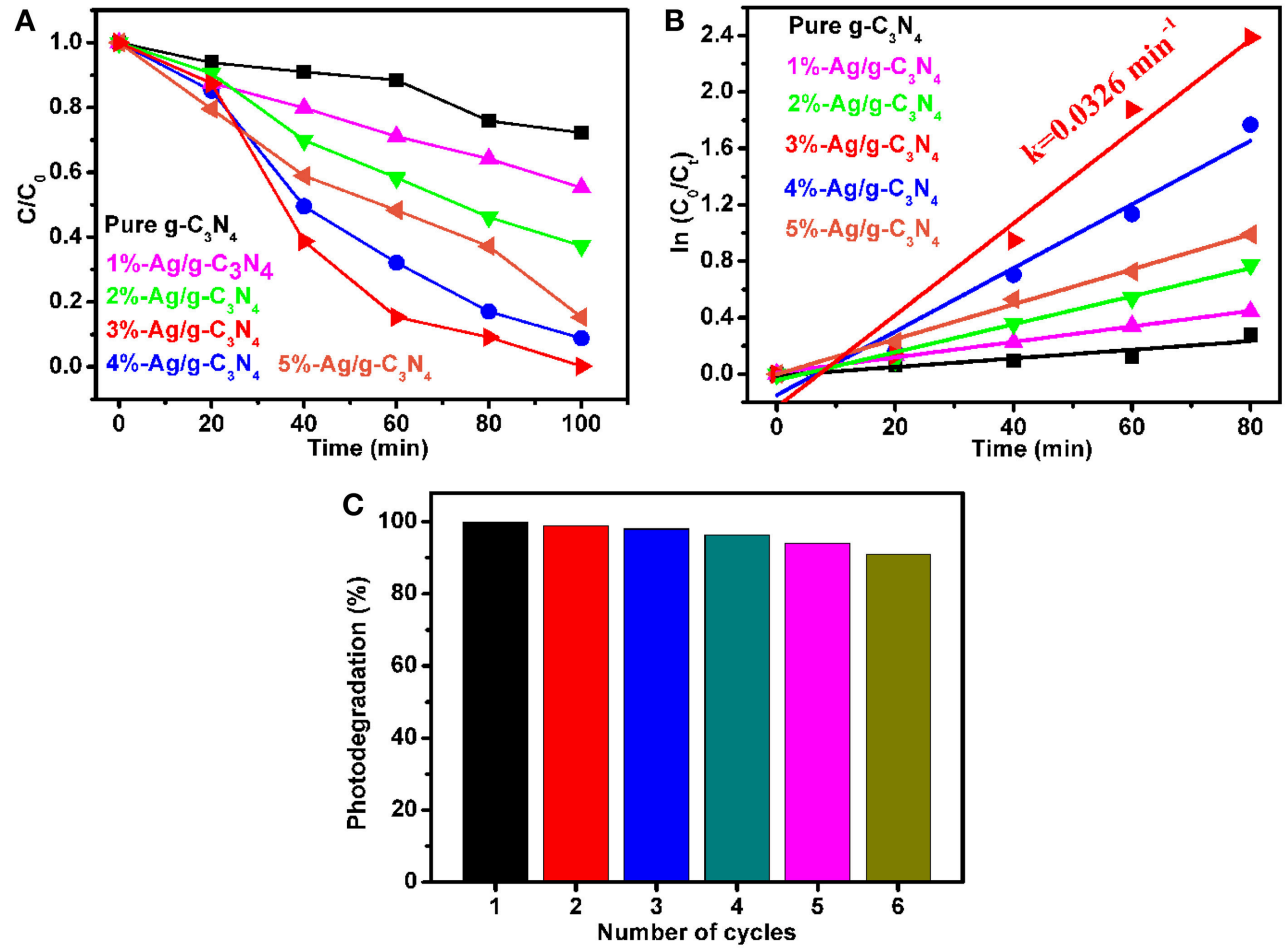
**(A)** photodegradation of RhB using pure g-C_3_N_4_, Ag/g-C_3_N_4_ with different Ag contents under simulated sunlight irradiation. **(B)** kinetic data for the degradation of RhB. **(C)** the photodegradation rate of RhB for six cycles using 3%-Ag/g-C_3_N_4_.

The photocatalytic activity of pure g-C_3_N_4_ and Ag/g-C_3_N_4_ for photodecomposition of RhB is tested under the simulated sunlight irradiation. As shown in Figure 7A, compared with that of pure g-C_3_N_4_, the Ag nanoparticle modified g-C_3_N_4_ shows an improved photocatalytic performance for decomposition of RhB aqueous. After irradiation for 100 min, the degradation of RhB is about 20% for pure g-C_3_N_4_ nanosheets and almost 100% for 3%-Ag/g-C_3_N_4_. Figure 7B shows the apparent reaction rate constant (k) of RhB photodegradation, which shows that the kinetic constant of 3%-Ag/g-C_3_N_4_ is almost 10 times higher than that of pure g-C_3_N_4_. When the mass ratio of Ag is in the range of 1–5 wt%, the enhanced photocatalytic activity is observed, due to effective enhanced the separation efficiency of photogenerated e^−^-h^+^ pairs at the Ag/g-C_3_N_4_ interface and the surface plasmon resonance (SPR) effect of Ag NPs (Duan et al., 2014), which are also supported by PL and UV–vis DRS results. Obviously, 3%-Ag/g-C_3_N_4_ has the highest photocatalytic activity, which may be due to that the excess Ag NPs will be working as recombination centers, or the active site on g-C_3_N_4_ surface is blocked (Ge et al., 2011). The photocatalytic activity of as-prepared Ag/g-C_3_N_4_ composites is compared to the typical photocatalysts including TiO_2_ and ZnO. The photocatalytic activity of 3%-Ag/g-C_3_N_4_ (*k* = 0.0326 min^−1^) in this work is higher that TiO_2_ (*k* = 0.0084 min^−1^) and ZnO (*k* = 0.0062 min^−1^) reported previously (Carvalho et al., 2015; Hao et al., 2019). In order to check the reusability of as-prepared Ag/g-C_3_N_4_ photocatalysts, the recycling test was carried out. As shown in Figure 7C, the photodegradation percentage of RhB is >90% after six cycles, which indicates the as-prepared photocatalysts owns a good stability. A gradually decreased photocatalytic activity is due to the loss of photocatalyst in the recovery process.

### Photoelectrochemical Performance

**Figure 8:**
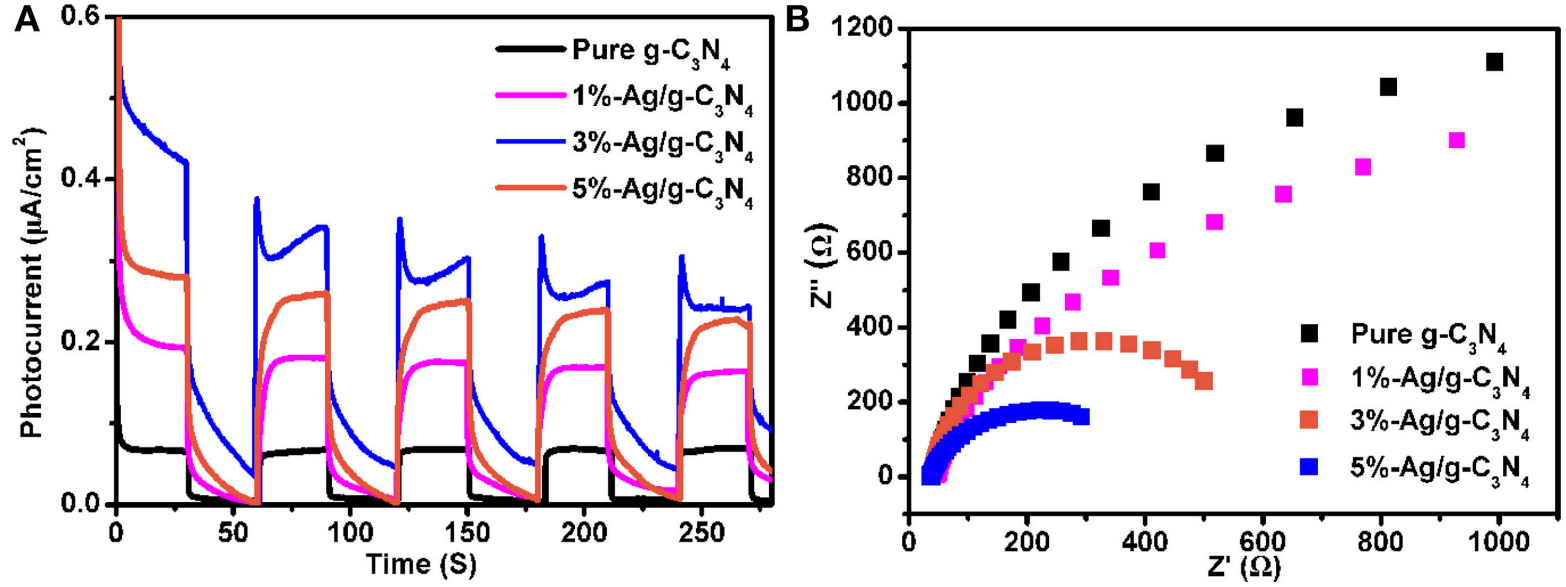
**(A)** photocurrent response and **(B)** electrochemical impedance spectroscopy of the pure g-C_3_N_4_, 1%-Ag/g-C_3_N_4_, 3%-Ag/g-C_3_N_4_, and 5%-Ag/g-C_3_N_4_ samples.

The charge separation efficiency is studied by using the photoelectrochemical measurements. Figure 8A shows that the photocurrent response of pure g-C_3_N_4_, 1%-Ag/g-C_3_N_4_, 3%-Ag/g-C_3_N_4_, and 5%-Ag/g-C_3_N_4_ samples under the simulated sunlight irradiation, which shows stable reproducible photocurrent responses over five on-off cycles. The photocurrent starts when the light was turned on, the photocurrent is close to zero when the light was turned off. The photocurrent density (0.50 μA/cm^2^) of 3%-Ag/g-C_3_N_4_ is bigger than the pure g-C_3_N_4_ (0.07 μA/cm^2^) and the 5%-Ag/g-C_3_N_4_ (0.38 μA/cm^2^). The stronger photocurrent is due to the higher separation efficiency of the photogenerated e^−^-h^+^ pairs of Ag/g-C_3_N_4_, which is consistent with its higher activity on photocatalytic decomposition of organic dyes.

In order to study the charge separation efficiency, the electrochemical impedance spectroscopy (EIS) Nynquist plots are used, and the EIS Nyquist plot of pure g-C_3_N_4_, 1%-Ag/g-C_3_N_4_, 3%-Ag/g-C_3_N_4_, and 5%-Ag/g-C_3_N_4_ sample is as shown in Figure 8B. Usually, the smaller the radius of EIS Nyquist plots is, the higher the separation efficiency of charge carriers is (Li et al., 2015). The radius on the EIS Nynquist plot of C_3_N_4_ is getting smaller after Ag modification, the order is 3%-Ag/g-C_3_N_4_ < 5%-Ag/g-C_3_N_4_ < 1%-Ag/g-C_3_N_4_ < g-C_3_N_4_, indicating that Ag modification indeed reduces the recombination of charge carries and increase the separation efficiency of photogenerated e^−^-h^+^ pairs, 3%-Ag/g-C_3_N_4_ is the best, which agrees well with PL spectra.

### Photocatalytic Mechanism

**Figure 9:**
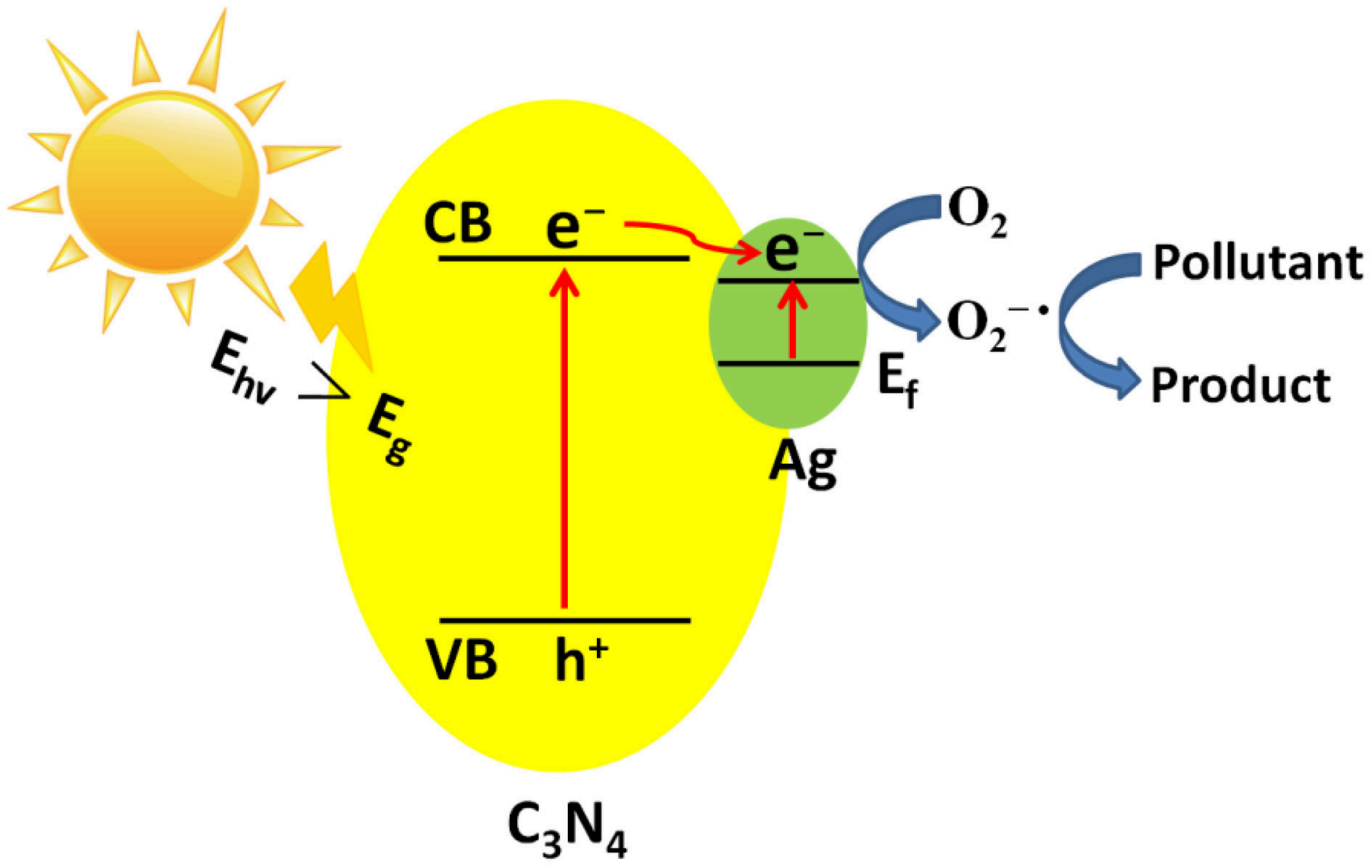
Photocatalytic mechanism of RhB decomposition over Ag/g-C_3_N_4_ composites.

Figure 9 shows the photocatalytic mechanism of Ag/g-C_3_N_4_ composites during RhB decomposition under sunlight irradiation. Ag nanoparticle modification enhances the photocatalytic performance of g-C_3_N_4_ due to the synergistic effect of two aspects, one is the SPR effect of metal Ag, another is the decrease of the recombination rate of photogenerated e^−^-h^+^ pairs (Ingram et al., 2011). When Ag/g-C_3_N_4_ is irradiated by the simulated sunlight irradiation, the e^−^-h^+^ pairs are separated, e^−^ is excited to CB of g-C_3_N_4_, h^+^ remains at VB of g-C_3_N_4_. Then e^−^ transfers to Ag NPs due to the high Schottky barrier of Ag, finally, transfers to the photocatalyst surface to join the reduction reaction. The generated e^−^ from two routes one from the plasmon excited Ag NPs and the other from the photoexcited g-C_3_N_4_ nanosheets. These e^−^ react with O_2_ to generate O^2−•^, and O^2−•^ radicals can discompose of RhB molecules to CO_2_ and H_2_O. Thus it is concluded that the adsorbed silver NPs have two functions, one is as the electron pool and the other is capture of the photoinduced electrons.

## Conclusion

In this work, Ag NPs modified g-C_3_N_4_ nanosheets are successfully prepared by a simple liquid-phase reduction method. In the Ag/g-C_3_N_4_ composites, the Ag NPs uniformly coasted on the g-C_3_N_4_ surface with the diameter range of 6~20 nm. After Ag loading, the Ag/g-C_3_N_4_ composites expand the visible light response and show an enhanced photocatalytic activity on RhB decomposition. The enhanced photocatalytic activity of Ag/g-C_3_N_4_ is due to the two reasons, one is the SPR effect of metal Ag, and another is the decrease of the recombination of the photogenerated e^−^-h^+^ pairs. Especially, 3%-Ag/g-C_3_N_4_ demonstrates the highest photocatalytic activity among the as-prepared samples for RhB decomposition, which is 10 times faster than the pure g-C_3_N_4_ nanosheet for decomposition of RhB. This work indicates that the Ag/g-C_3_N_4_ photocatalyst is one of promising candidates to treat organic pollutants in the waste water.

## Author Contributions

All authors listed have made a substantial, direct and intellectual contribution to the work, and approved it for publication.

### Conflict of Interest Statement

The authors declare that the research was conducted in the absence of any commercial or financial relationships that could be construed as a potential conflict of interest.
